# Milk and Tuberculosis

**Published:** 1911-12-23

**Authors:** 


### MILK AND TUBERCULOSIS.

The interesting report presented before the Public Health Committee of the London County Council upon the results obtained by the inoculation of 9,000 guinea-pigs with sediment from milk affords a valuable illustration of the necessity of approaching any important question from all possible avenues of investigation. The discovery of the fact that cows suffer from tuberculosis led at once to the assumption that milk from such cows is a source of great danger to man. A bacteriologist or a morbid anatomist from time to time raised a protest against too great stress being laid upon milk as a source of infection in tuberculosis, but so deep was the conviction as to danger in the minds of the majority of medical men interested in the subject, that when Koch expressed a contrary opinion the profession refused to accept his belief.

However just in his views the scientist may endeavour to be he, unfortunately, no less than other men, cannot fail to possess mental bias, and the feeling being so strong upon the subject, it appeared that before commissions were appointed there would be little doubt as to their findings. We have heard comparatively recently the conclusions at which one commission has arrived. To those who originally possessed doubts these conclusions were not very convincing. They depended largely upon the recognition of two types of bacilli. In such a delicate matter as this there are many sources of error. To base extremely important conclusions upon slight differences of bacilli would appear to be scarcely warranted by the teachings of bacteriology. Whatever opinion, however, may be held upon such a point as this, few bacteriologists or morbid anatomists doubted that if milk were inoculated into guinea-pigs and those guinea-pigs died of tuberculosis, the fatal result was an evidence that the disease tuberculosis had been transferred from the cow to the guinea-pig. Now, however, Mr. Reynolds presents a report as a result of extensive investigations, which shows that the milk of healthy cows produces as serious results in guinea-pigs as the milk of cows suffering from tuberculosis. Such a conclusion, if confirmed by other observers, may be said to reopen the whole question of the danger to man of infection from tuberculosis through cow's milk.

It may be interesting in this connection to mention that some medical men practising in China state that the same forms of tuberculosis which occur in children in this country affect the native children there. Yet while this is true no cow's milk is given to the children. The cows which the Chinese possess are not kept for the purpose of providing milk, but are used in agricultural work as draught animals.

